# Establishment and pathophysiological evaluation of a novel model of acute compartment syndrome in rats

**DOI:** 10.1186/s12891-024-07187-6

**Published:** 2024-01-17

**Authors:** Qi Dong, Yubin Long, Lin Jin, Guanlin Hou, Guoqiang Li, Tao Wang, Huiyang Jia, Yingchao Yin, Junfei Guo, Huijie Ma, Sujuan Xu, Yingze Zhang, Zhiyong Hou

**Affiliations:** 1 Department of Orthopaedics Surgery, Third Hospital of Hebei Medical University, Shijiazhuang, 050051 China; 2 Orthopaedic Research Institute of Hebei Province, Third Hospital of Hebei Medical University, Shijiazhuang, China; 3 Department of Nephrology, Third Hospital of Hebei Medical University, Shijiazhuang, China; 4 Hebei Medical University, Shijiazhuang, China; 5 Department of Orthopaedics Surgery, Baoding No.1 Central Hospital, Baoding, China; 6 WLSA Shanghai Academy, Shanghai, China; 7 Department of Joint Surgery, Honghui Hospital, Xi’an Jiaotong University, Xi’an, China

**Keywords:** Acute compartment sundrome, Animal models, ICP

## Abstract

**Background:**

Researches have used intra-compartmental infusion and ballon tourniquest to create high intra-compartmental pressure in animal models of Acute Compartment Syndrome (ACS). However, due to the large differences in the modeling methods and the evaluation criteria of ACS, further researches of its pathophysiology and pathogenesis are hindered. Currently, there is no ideal animal model for ACS and this study aimed to establish a reproducible, clinically relevant animal model.

**Methods:**

Blunt trauma and fracture were caused by the free falling of weights (0.5 kg, 1 kg, 2 kg) from a height of 40 cm onto the lower legs of rats, and the application of pressures of 100 mmHg, 200 mmHg, 300 mmHg and 400 mmHg to the lower limbs of rats using a modified pressurizing device for 6 h. The intra-compartmental pressure (ICP) and the pressure change (ΔP) of rats with single and combined injury were continuously recorded, and the pathophysiology of the rats was assessed based on serum biochemistry, histological and hemodynamic changes.

**Results:**

The ΔP caused by single injury method of different weights falling onto the lower leg did not meet the diagnosis criteria for ACS (< 30 mmHg). On the other hand, a combined injury method of a falling weight of 1.0 kg and the use of a pressurizing device with pressure of 300 mmHg or 400 mmHg for 6 h resulted in the desired ACS diagnosis criteria with a Δ*P* value of less than 30 mmHg. The serum analytes, histological damage score, and fibrosis level of the combined injury group were significantly increased compared with control group, while the blood flow was significantly decreased compared with control group.

**Conclusion:**

We successfully established a new preclinical ACS-like rat model, by the compression of the lower leg of rats with 300 mmHg pressure for 6 h and blunt trauma by 1.0 kg weight falling.

## Introduction

Acute compartment syndrome (ACS) is a serious complication in traumatic orthopedics, which is defined as a series of syndromes caused by progressive neuromuscular injury caused by high pressure in a closed compartment surrounded by bone and fascia [1]. With the rapid development of society and the frequent occurrence of high-energy and/or multiple injuries, the incidence of ACS is increasing annually. According to statistics, the incidence of ACS is about 3.1 per 100,000, and the male to female ratio is 10:1 [2]. Increased compartment content and decreased compartment volume caused by various risk factors may cause hypoperfusion and microcirculation disturbance in the involved compartment tissue, leading to irreversible necrosis of the limb, or even life-threatening consequences [2]. Although the disease was first described 130 years ago, its diagnosis and treatment remain challenging [3].The uncertainty of the pathophysiology of ACS occurrence and development limits its clinical diagnosis and treatment, and the underlying mechanisms of ACS occurrence and development will be the future focus of the disease research.

Due to the limitations to the clinical studies on ACS patients, animal models are important research tools. Many studies have suggested that fascial compartment hypertension is the initiation factor of ACS. The classical ACS animal model was created by injection of fluid into the fascia compartment to generate high pressure [4–9]. However, due to the different physiological characteristics of fascia in different animals, the fascia compartment pressure is often variable in the injection models [10]. Alternatively, some researchers placed a balloon into the fascia compartment and inflated the balloon to increase the pressure in the fascia compartment to simulate ACS [1, 11–14]. However, these two animal models only considered the effect of compartment pressure on tissues. In addition to intra-compartmental inflation, ischemia-reperfusion injury caused by cuff compression or vascular ligation is the common methods to induce ACS in animal models [15–19]. However, Heppenstall et al. indicated that ischemic injury caused by tourniquet or ligation is not suitable for simulating ACS [20]. Furthermore, some studies have found that trauma is an important factor for the occurrence of ACS [21, 22], and about 70% of trauma-induced ACS patients are related to fracture [23]. Following fracture with soft tissue injury, the tough fascia along with swollen tissue increased pressure in the fascia compartment, resulting in reduced tissue perfusion and irreversible tissue damage [2, 24]. It is highly likely that the initial trauma contributes to the physiological changes of the fascial compartment, including inflammation and microvascular injuries, leading to symptoms of ACS. Due to the elasticity of the animal fascia, the increase in compartment pressure after trauma is not obvious [18]. However, there are great variances in the establishment methods and evaluation criteria for ACS animal models. As a result, these models cannot fundamentally simulate the pathophysiological processes of clinical ACS, which brings great challenges to further study on the pathogenesis of ACS.

In this study, we try to create a novel and replicable ACS animal model similar to the pathophysiology of clinical observations of ACS, through which the molecular mechanisms and pathophysiology of ACS can be further studied, in order to improve the diagnosis and treatment of ACS.

## Methods

### Animals

All experiments were approved by the Animal Care and Use Committee of the Third Hospital of Hebei Medical University (S2020-022-1) and were performed in accordance with the National Institutes of Health Guidelines for the Care and Use of Laboratory Animals. Furthermore, all experiments were compliant with NIH guidelines for the humane care and use of laboratory animals. A total of seventy SD rats (12-weeks-old, healthy, male, 290-310 g) were purchased from the Beijing Vital River Laboratory Animal Technology Co. (Beijing, China). Rats were maintained on standard chow and housed under 12-hour light/12-hour dark cycles, controlled temperature (22-24 °C) and humidity (50–65%). All experiments were performed at similar times of the day to negate any circadian rhythm effects on the rats.

### Establishment of the blunt injury animal model

Twenty SD rats were randomly assigned to blunt trauma experiment, with five rats in each group: control (only anesthesia and fixation in the compression device without compression), 0.5 kg, 1.0 kg, and 2.0 kg blunt trauma group. SD rats were anesthetized with 3% isoflurane for induction and then maintained with 1.5% isoflurane. After shaving the lower limb region, the right proximal tibia of each rat was half exposed, and blunt injury was made using a custom-made device. Different weights (0.5 kg, 1 kg, 2 kg) falling freely from a height of 40 cm onto the lower leg of rats was used to cause different degrees of trauma, as described by Altay et al. previously [25]. And the pressure inside the anterior tibial fascia compartment and the blood pressure were then continuously measured to determine the effects of injury by different weights. Postoperative analgesia was administered with 1% lidocaine infiltration and continued until sampling time.

### Establishment of novel ACS animal model by combined blunt and compression injuries

A total of fifty SD rats were randomly assigned to the combined injuries group. Twenty five rats were randomly assigned to the 3-day euthanized group, and the other twenty five rats in 14-day euthanized group. In the sampling group, five rats were randomly chosen in each compression pressure group (100 mmHg, 200 mmHg, 300 mmHg, and 400 mmHg) and the control group. And SD rats were anesthetized with 3% isoflurane for induction and then maintained with 1.5% isoflurane. Blunt trauma was caused using a custom-made device. Blunt trauma was created by free falling of a weight of 1.0 kg onto the lower leg from a height of 40 cm. Three hours after the blunt trauma, a novel compression device consisting of a pressure gauge cuff (DRO-SX, Constant Hui Medical Instruments LTD.) was embedded into a rigid plastic tube. The lower leg was compressed by the device for 6 h at one of the following pressures: 100 mg, 200 mg, 300 mg and 400 mg each. Postoperative analgesics 1% lidocaine were administered and continued until the time of sampling.

The sample size of the experiment was estimated by results from the previous preliminary experiment. After the experiment all animals were euthanized strictly in accordance with AVMA Guidelines for the Euthanasia of Animals: 2020 Edition*. To be precise the rats were euthanized by peritoneal injection of.

200 mg/kg sodium pentobarbital and 10 mg/mL lidocaine peritoneal injection. All animals were anaesthetized and unconscious during euthanasia, and all efforts were made to minimize animal suffering and to reduce the number of animals used. Subsequently all animal carcasses were burned in a unified manner.

### Xray examination

After anesthesia, the rats were placed in the prone position. And an anteroposterior X-ray of the tibia of the rats after blunt injury was performed by a GE 2000D mammography device to confirm fracture status. The exposure conditions were 52 kV and 4.10 mAs.

### Compartmental pressure measurement

Dynamic pressure measurement was performed using an automatic fascial pressure measuring instrument (CYY-1, Liyang Wanda Electronics Co. LTD.). CYY-1 consists of a recording box, switch, numerical display, and a single-use set comprising a syringe of physiological saline and a pressure transducer. The product instructions were closely followed to apply the device to the experiment animals. In detail, first follow the product instructions to connect. Once started, the air was automatically vented, and the needle was placed at the same height of the pressure measuring limb to complete the pressure measurement calibration. After anesthesia, the six-gauge needle was inserted into the anterior tibial fascia compartment of the rats to a depth of 10 mm, and the pressure was measured by pressing the start button. After the pressure was stable, the pressure was measured thrice continuously and the average values were recorded. Each rat was measured every 6 h for 3 days and pressure values were recorded.

### Blood pressure measurement

Blood pressures were measured by Panlab NIBP System (Harvard Apparatus). The rats were lightly fixed in fixator and placed in a 37℃ preheated bath for 30 min. Blood pressure was measured when the tail vessels were dilated and the pulse wave was stable. Tail arterial blood pressure was measured and averaged at least three times for each rat, with an interval of more than 5 min between each measurement.

### Histology staining

Animals were euthanized after experiments, and tibialis anterior (TA) muscles was harvested and fixed in 10% neutral buffered formalin (P110, Solarbio, China) for 48 h before being transferred to 70% ethanol (Yongda Chemical Reagent Company, China). TA muscles were embedded in paraffin and sliced into 4 μm-thick sections and stained with hematoxylin and eosin (H&E) or Masson’s trichrome stains.

The detailed H&E staining steps were as follows:1. Slides were successively transferred into staining jars with xylene (Yongda Chemical Reagent Company, China) for 3 min, 100% ethanol for 1 min, 90% ethanol for 20 s, and 70% ethanol for 20 Sect. 2. The slides were incubated with hematoxylin solution (P1120, Solarbio, China) in a staining jar for 10 min to stain the nuclei. 3. The slides were transferred to a staining jar with Eosin solution (P1120, Solarbio, China) for 3 min. 4. The slides were then subsequently transferred into staining jars with 70% ethanol for 20 s, 90% ethanol for 20 s, 100% ethanol for 1 min and xylene for 3 min. 5. The slides were then taken out from xylene and placed in a fume hood till the slides were dry. 6. The samples were mounted with xylene-based mounting media and covered with cover slides. Clips were used to press the slides to squeeze bubbles.

The procedure of Masson’s trichrome staining (G1340, Solarbio, China) were as follows: 1. The slides were transferred into staining jars with xylene for 3 min, 100% ethanol for 1 min, 90% ethanol for 20 s, and 70% ethanol for 20 Sect. 2. The slides were transferred into staining jars with bouin solution 10 min, harris hematoxylin for 3 min, water for 2 min, 0.5% hydrochloric acid alcohol for 20s, water for 5 min, masson composite dyeing solution for 5 min, 0.2% acetic acid aqueous solution for 30s, 5% phosphomolybdic acid for 10 min, 0.2% acetic acid aqueous solution for 30s, 2% aniline blue solution for 30s, and anhydrous ethanol for 3 min. 3. The slides were transferred into staining jars with 70% ethanol for 20 s, 90% ethanol for 20 s, 100% ethanol for 1 min and xylene for 3 min. 4. The slides were taken out from xylene and placed in a fume hood till the slides were dry. 5. The samples were mounted with xylene-based mounting media and covered with cover slides. Clips were used to press the slides to squeeze bubbles.

H&E and Masson’s trichrome stained image were captured with a Leica DMI 6000B microscope. H&E staining was performed to evaluate the pathological damage to skeletal muscle. Five random fields were selected from each sample, as described previously [26]. Histological damage score was performed as follows: disorganization and degeneration of the muscle fibers (0: Normal, 1: Mild, 2: Moderate, 3: Severe); and inflammatory cell infiltration (0: Normal, 1: Mild, 2: Moderate, 3: Severe). Masson’s trichrome staining was performed to measure skeletal muscle fibrosis after combined injury, and the fibrotic area was quantified for 5 random fields. Samples were randomized and graded by two independent blind examiners. Discrepancies in scoring were resolved by discussion, with a third examiner being consulted when consensus could not be reached.

### Serum analysis

Serum creatine kinase (CK), lactate dehydrogenase (LDH), creatine kinase isoenzyme MB (CKMB), alanine transaminase (ALT), aspartate transaminase (AST), urea nitrogen and creatinine were measured using the AU5800 instrument (Beckman Coulter, Brea, CA, USA). Blood lactate (LAC) was determined using Vitro 4600 chemistry analyzer (Ortho Clinical Diagnostics Inc, USA).

### Blood flow measurement

The Vevo 2100 ultrasound system (Visual Sonics, Toronto, Canada) and a 40 MHz high-frequency linear array probe (MS550D, Visual Sonics, Toronto, Canada) were used to measure blood flow-velocity. The rats were anesthetized with oxygen inhalation of 1.5% isoflurane, and then the rats were immobilized on a heating pad in the supine position. After anesthesia, animal body temperature was maintained by heating pads. An equilibration time of 10 min was given in order to ensure stable body temperature and heartbeat. Image acquisition was initiated with an MD550 transducer probe placed along the tibial arteriovenous region to obtain the long axis view. Then, the Color Doppler images were captured to further confirm tibial arteriovenous and blood flow was examined in the pulsed-wave Doppler mode. The arteries were too small for accurate diameter measurements, so only blood flow velocity was reported. After image acquisition, Vevo workstation was used to evaluate and analyze the acquired images. RI = (PSV-EDV)/PSV, where RI = resistive index, PSV = peak systolic velocity, and EDV = end diastolic velocity.

### Statistical analysis

All data was shown as means ± SD and was analyzed using the SPSS 13.0 statistical software (SPSS Inc., Chicago, IL, USA). Shapiro-Wilk test was used to evaluate the normal distribution. The unpaired two-tailed student’s t-tests were used to analyze for significant differences between any 2 groups. Statistical analysis among multiple groups was performed using one-way analysis of variance (ANOVA) followed by post-hoc LSD-t-tests. A *P*-value < 0.05 was considered as significantly different.

## Results

### Blunt trauma alone could not induce rats ACS model

**Fig. 1:**
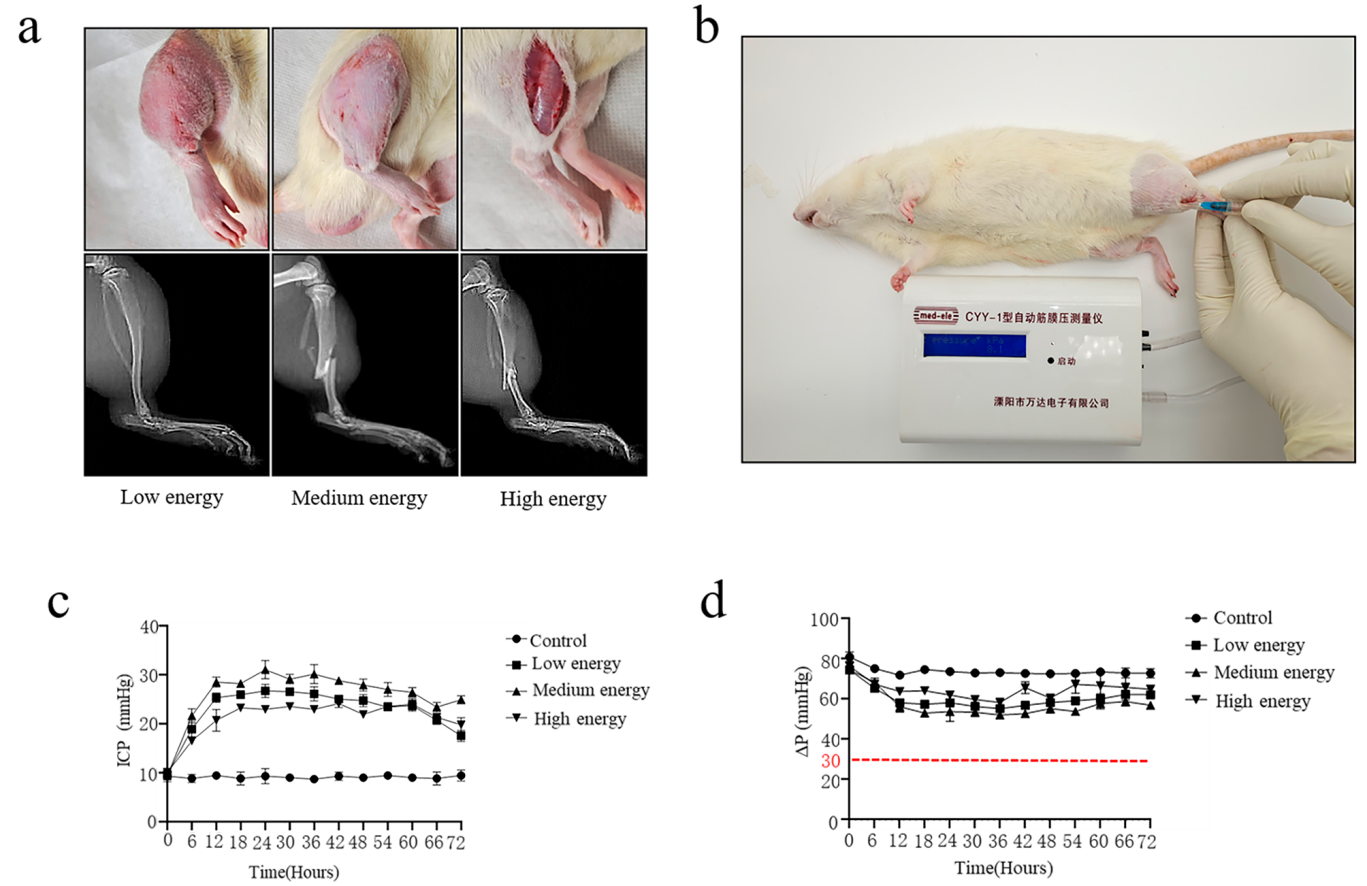
Effects of simple blunt trauma on the lower leg of rats. (**a**) Tissue morphology and X-ray images of the lower legs of rats after different degrees of blunt trauma. (*n* = 5 for each individual group). (**b**) ICP of the lower legs of rats measured by manometry. (**c**) Rats’ ICP levels monitored using manometry for 72 h. (*n* = 5 for each individual group). (**d**) Rats’ ΔP levels for 72 h. Low energy 0.5 kg group; Medium energy 1.0 kg group; High energy 2.0 kg group; ICP, intra-fascial pressure; ΔP, Diastolic pressure-intra-fascial pressure; kg, kilogram. (*n* = 5 for each individual group)

Free falling different weights (0.5 kg, 1 kg, 2 kg) free falling onto the lower leg of rats were used to investigate whether blunt trauma alone could cause ACS [25]. As shown in Fig. 1a, the lower leg was significantly swollen for 3 h after impact by low energy 0.5 kg weight. The lower leg was swollen and fractured after medium energy 1.0 kg weight impact. Open injury and fracture of the lower leg was caused by the use of high energy 2.0 kg weight. The pressure inside the anterior tibial fascia compartment was continuously measured (Fig. 1b). Previous studies reported that intra-compartmental pressure (ICP) was an effective auxiliary method for the diagnosis of ACS, and the difference between diastolic and intra- compartmental pressure (ΔP) of < 30 mmHg was the gold standard for the diagnosis of ACS [27–29]. Our results showed that the ICP in the different energy groups were significantly higher than that of control group (Fig. 1c; Table 1). However, none of the three energy groups had Δ*P* values of less than 30mmHg (Fig. 1d; Table 1), which indicated that blunt trauma alone could not successful induce ACS animal model.

**Table 1:** Summary of ICP and ΔP after blunt injury

Variable	Control	0.5 kg	1 kg	2 kg
0 h (mmHg)	9.300 ± 1.137, **75.58 ± 3.966**	9.660 ± 0.5672 ^NS^, **77.42 ± 5.530** ^NS^	9.450 ± 0.8551 ^NS^, **73.25 ± 4.204** ^NS^	10.05 ± 0.8551 ^NS^, **72.05 ± 4.385** ^NS^
6 h (mmHg)	8.850 ± 0.8216, **73.89 ± 2.144**	18.90 ± 1.626 ^*^, **65.20 ± 3.771** ^*^	21.60 ± 1.443 ^*,^ **60.50 ± 5.507** ^*^	16.50 ± 0.5303 ^*^, **65.60 ± 4.902** ^*^
12 h (mmHg)	9.450 ± 0.6708, **75.43 ± 3.602**	25.35 ± 0.9779 ^*^, **59.93 ± 2.951** ^*^	28.50 ± 1.061^*^ **57.60 ± 3.691** ^*^	20.70 ± 2.225 ^*^, **61.40 ± 4.764** ^*^
24 h (mmHg)	9.300 ± 1.555, **76.06 ± 6.257**	26.70 ± 1.362 ^*^, **56.00 ± 2.731** ^*^	31.05 ± 1.882 ^*^, **55.05 ± 3.169** ^*^	22.95 ± 0.6708 ^*^, **60.75 ± 1.245** ^*^
48 h (mmHg)	9.000 ± 0.5303, **76.68 ± 5.113**	24.75 ± 1.186 ^*^, **57.95 ± 2.929** ^*^	27.90 ± 1.232 ^*^, **58.00 ± 4.033** ^*^	21.90 ± 0.3354 ^*^, **60.20 ± 4.400** ^*^
72 h (mmHg)	9.450 ± 1.137, **76.21 ± 4.228**	17.55 ± 1.137 ^*^, **65.35 ± 2.756** ^*^	24.90 ± 0.8216 ^*^, **61.20 ± 4.629** ^*,^	19.80 ± 1.462 ^*^, **62.30 ± 3.064** ^*^

Values are presented as mean ± SD. ^*^P < 0.0.5, compared with control group. h, hour; ICP, intra-fascial pressure; SD, standard deviation; NS, not significance; The data of ΔP is in bold font

### The effect of combined injury in ACS formation

**Fig. 2:**
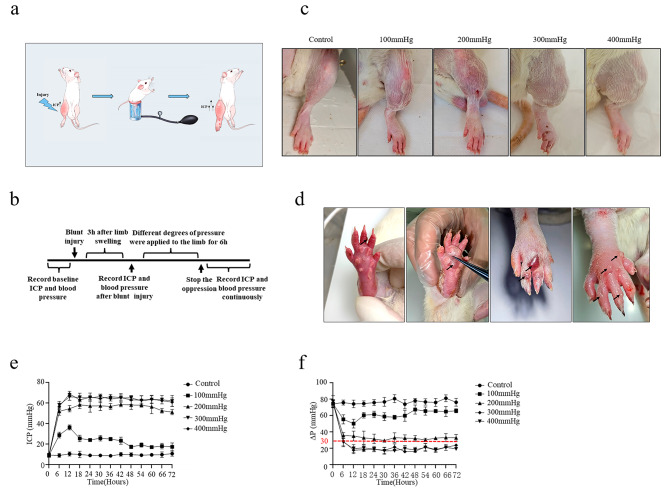
Effects of combined injury on the lower legs of rats. (**a**) Schematic diagram of processes for ACS model. (**b**) Flow chart of the experimental design for ICP. (**c**) Tissue morphology of the lower legs. (*n* = 5 for each individual group). (**d**) Tension blisters of the lower legs in rats. (**e**) Rats’ ICP levels monitored using manometry for 72 h. (*n* = 5 for each individual group). (**f**) Rats’ ΔP levels for 72 h. ICP, intra-fascial pressure; ΔP, Diastolic pressure-intra-fascial pressure; kg, kilogram. (*n* = 5 for each individual group)

This study had demonstrated that different degrees of blunt trauma did not lead to compartment syndrome-like hypertension combined with ischemic injury in the rat lower leg, which might be due to the strong extensibility of the rat fascia. To further develop a novel method to induce ACS, a specific compression device was used to simulate fascia constraint and compression of muscles. The ICP measurements were shown in Fig. 2a and b, respectively. The ICP results from different compression pressures (100 mmHg, 200 mmHg, 300 mmHg, 400 mmHg) for 6 h after moderate energy blunt trauma were shown in Table 2. As expected, increased pressure followed by compression release of the lower legs for 24 h led to significantly swollen rat calves (Fig. 2c). Interestingly, some tension blisters on the feet and ankles of rats after the 300 mmHg and 400 mmHg compression were observed, which might be due to excessive swelling of the lower legs (Fig. 2d). Furthermore, the ICP in different groups were significantly higher compared to the control group, especially in the 200 mmHg, 300 mmHg and 400 mmHg compression groups (Fig. 2e; Table 2). Encouragingly, the Δ*P* values in the 300 mmHg and 400 mmHg compression groups were less than 30 mmHg (Fig. 2f; Table 2), meeting the criteria for ACS diagnosis. Taken together, these data demonstrated that combined injury of moderate blunt trauma and outside compression could lead to delta *P* values of less than 30 mmHg, meeting the diagnosis criteria of ACS, and the blister formation was also consistent with clinical symptoms of ACS.

**Table 2:** Summary of ICP and ΔP after combined injury

Variable	Control	100 mmHg	200 mmHg	300 mmHg	400 mmHg
0 h (mmHg)	9.450 ± 0.6708, **74.55 ± 5.408**	9.15 ± 0.6275 ^NS^, **74.71 ± 4.121** ^NS^	8.850 ± 0.6275 ^NS^, **76.01 ± 3.718** ^NS^	9.600 ± 1.232 ^NS^, **77.24 ± 7.224** ^NS^	9.450 ± 0.6708 ^NS^, **75.05 ± 4.119** ^NS^
6 h (mmHg)	9.100 ± 1.773, **75.98 ± 3.081**	28.95 ± 2.836 ^*^, **55.55 ± 5.575** ^*^	52.05 ± 3.846 ^*^, **35.81 ± 3.518** ^*,^	58.35 ± 3.412 ^*^, **27.73 ± 4.604** ^*^	56.85 ± 2.826 ^*^, **28.79 ± 6.574** ^*,^
12 h (mmHg)	10.58 ± 1.985, **74.34 ± 4.659**	36.30 ± 2.407^*^, **50.32 ± 5.419**	54.60 ± 3.155 ^*^, **35.22 ± 5.920** ^*^	66.15 ± 3.329 ^*^, **19.83 ± 4.977** ^*,^	69.00 ± 2.543 ^*^, **17.86 ± 4.255** ^*^
24 h (mmHg)	9.09 ± 0.9350, **75.93 ± 3.594**	24.15 ± 1.443 ^*^, **61.53 ± 3.860** ^*^	57.30 ± 6.084 ^*^ **31.63 ± 5.786** ^*^	65.25 ± 2.187 ^*^, **19.29 ± 3.338** ^*,^	65.85 ± 4.018 ^*^, **19.24 ± 2.359** ^*^
48 h (mmHg)	9.800 ± 1.866, **78.32 ± 2.794**	17.40 ± 1.791 ^*^, **67.47 ± 5.596** ^*^	58.20 ± 4.456 ^*^ **32.22 ± 3.630** ^*^	65.10 ± 2.275 ^*^, **18.34 ± 3.128** ^*^	63.15 ± 4.189 ^*^, **16.55 ± 0.818** ^*^
72 h (mmHg)	10.81 ± 2.502, **73.67 ± 4.977**	17.40 ± 3.802 ^*^, **69.96 ± 5.308** ^*^	51.30 ± 2.576 ^*,^ **32.95 ± 4.060** ^*^	62.10 ± 5.393 ^*^, **19.43 ± 0.775** ^*^	61.05 ± 4.162 ^*^, **23.88 ± 1.559** ^*^

Values are presented as mean ± SD. * *P* < 0.0.5, compared with control group. h, hour; ICP, intra-fascial pressure; SD, standard deviation; NS, not significance. The data of ΔP is in bold font

### Biochemical and histological changes caused by combined injury induced ACS formation

**Fig. 3:**
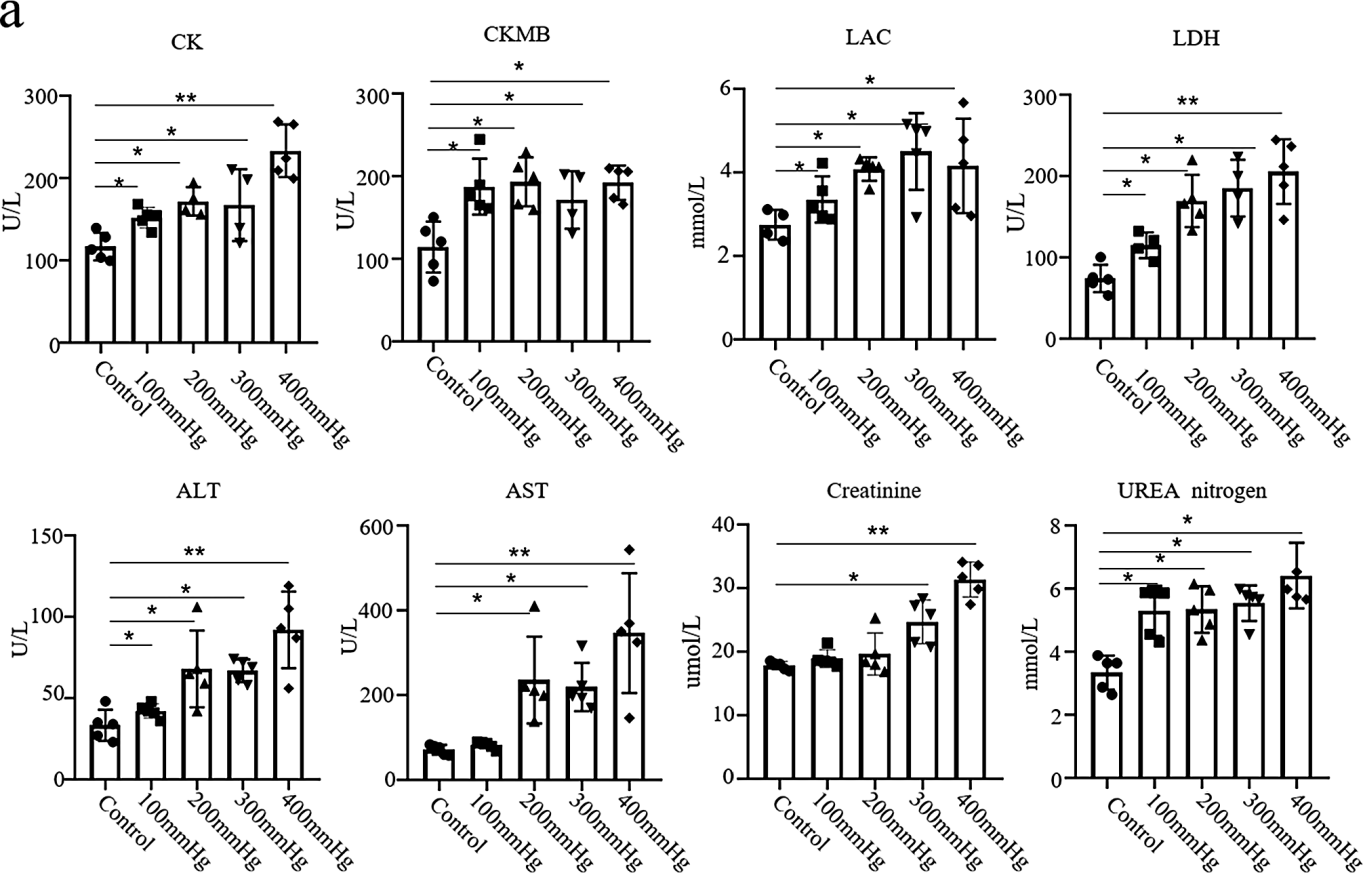
Biochemical examination of combined injury on the lower leg of rats. (**a**) Serum CK, CKMB, LAC, LDH, ALT, AST, creatinine and urea nitrogen evaluated 3 days after different degrees of combined injury. (*n* = 5 for each individual group). Data are presented as mean ± SD. *P < 0.05, **P < 0.01 versus with control group. CK, creatine kinase; CKMB, creatine kinase isoenzyme MB; LAC, lactate; LDH, lactate dehydrogenase; ALT, alanine transaminase; AST, aspartate transaminase; SD, standard deviation

To further assess the damage caused by combined injury in rats, the biochemical changes related to combined injury associated ACS formation were investigated. As shown in Fig. 3a, serum CK, LAC, LDH, ALT, AST, creatinine and urea nitrogen increased significantly in positive relationship to the increase in compression pressure. However, serum CKMB Values In the compression groups were higher than the control group, but remained at the same level when the compression pressure increased.

**Fig. 4:**
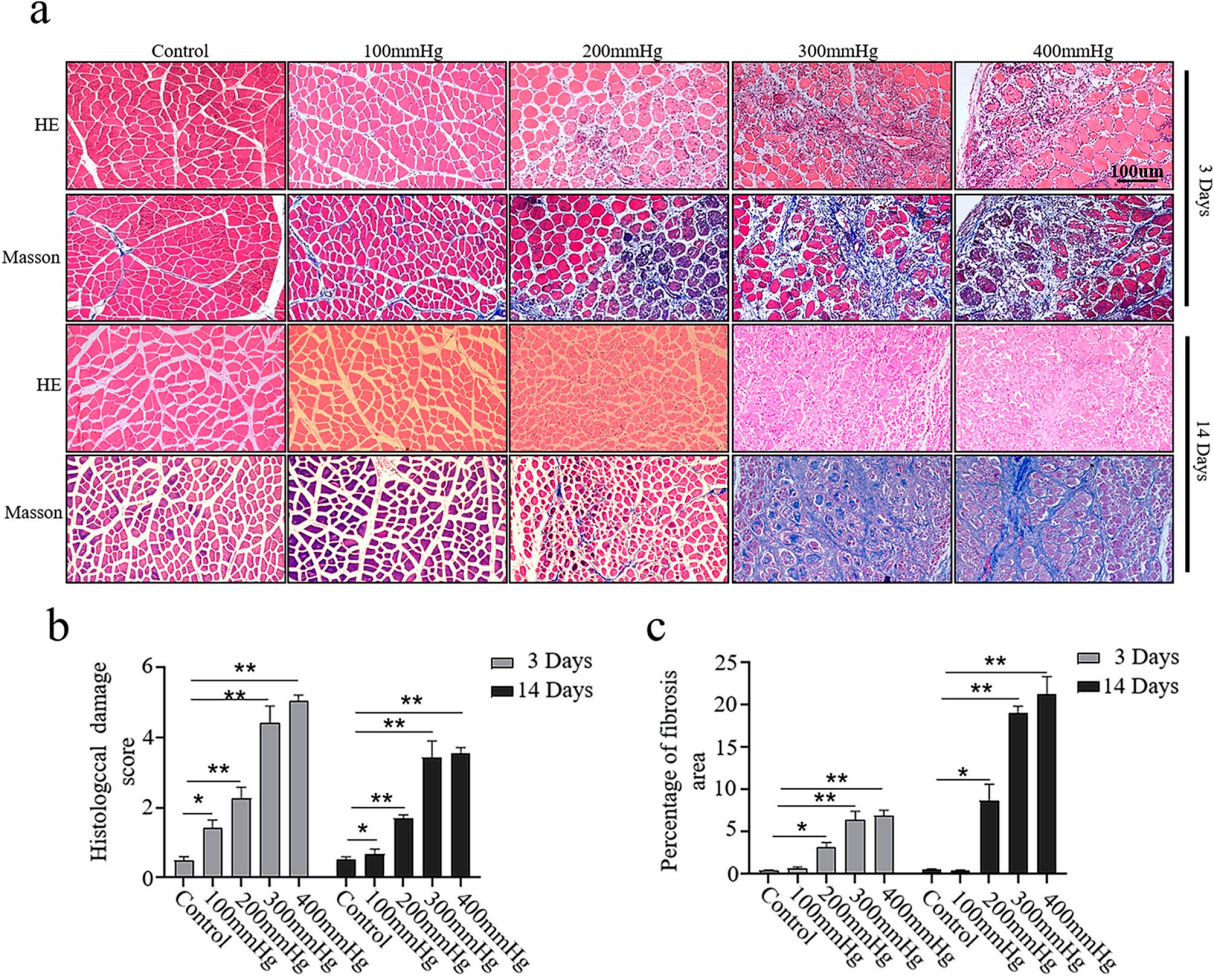
Histological staining of the lower legs of rats following combined injury. (**a**) Representative H&E or Masson’s trichrome images of tibialis anterior muscles 3 or 14 days after different degrees of combined injury. (*n* = 5 for each individual group). (**b**) Injury score analyzed on H&E sections. (*n* = 5 for each individual group). (**c**) Fibrosis analyzed with Masson’s trichrome stain. (*n* = 5 for each individual group). Data are presented as the mean ± SD. *P < 0.05, **P < 0.01 versus with control group. H&E, hematoxylin and eosin; SD, standard deviation

Hypoxia and trauma in the early stage of ACS lead to myocyte edema, necrosis and inflammation. Myocyte necrosis that cannot be repaired will eventually lead to muscle fibrosis and muscle contracture eventually. As shown in Fig. 4a, H&E staining 3 days after combined injuries indicated that the muscle fibers in the 100 mmHg combined injuries group were slightly edematous, and the muscle fibers were moderately edematous with inflammatory infiltration in 200 mmHg combined injuries group. Moreover, severely disorganized edematous muscle fibers and large amount of inflammatory infiltration were observed in 300 mmHg and 400 mmHg combined injuries groups.

After the acute trauma, the results showed that the muscle fibers edema subsided 14 days after the 300 mmHg and 400 mmHg combined injury. However, there were still large numbers of inflammatory cells infiltration. In addition, parts of muscle fibers were phagocytosed and decomposed by inflammatory cells gradually, and the muscle fibers in the 300 mmHg and 400 mmHg combined injuries groups basically returned to normal. The histological injury scores were shown in Fig. 4b. As shown in Fig. 4a, a small amount of disordered extracellular matrix was deposited among the muscle fibers 3 days after combined injury in the 300 mmHg and 400 mmHg combined injury group. Furthermore, most of the muscle fibers atrophied and were replaced by extracellular matrix that was dense, scattered and irregular, and some were observed as clumps 14 days after the 300 mmHg and 400 mmHg combined injuries. However, there was no extracellular matrix deposition in the muscle space of the 300 mmHg combined injury group and a small amount was found in the 400mmHg combined injury group. The percentage of extracellular matrix deposition was significantly increased in the 300 mmHg and 400 mmHg combined injury group compared with the 200 mmHg combined injury group (Fig. 4c). Collectively, these data indicated that histological injury and extracellular matrix deposition are associated with the development of combined injury-induced ACS.

### The evaluation of lower limb blood flow in combined injury induced ACS

**Fig. 5:**
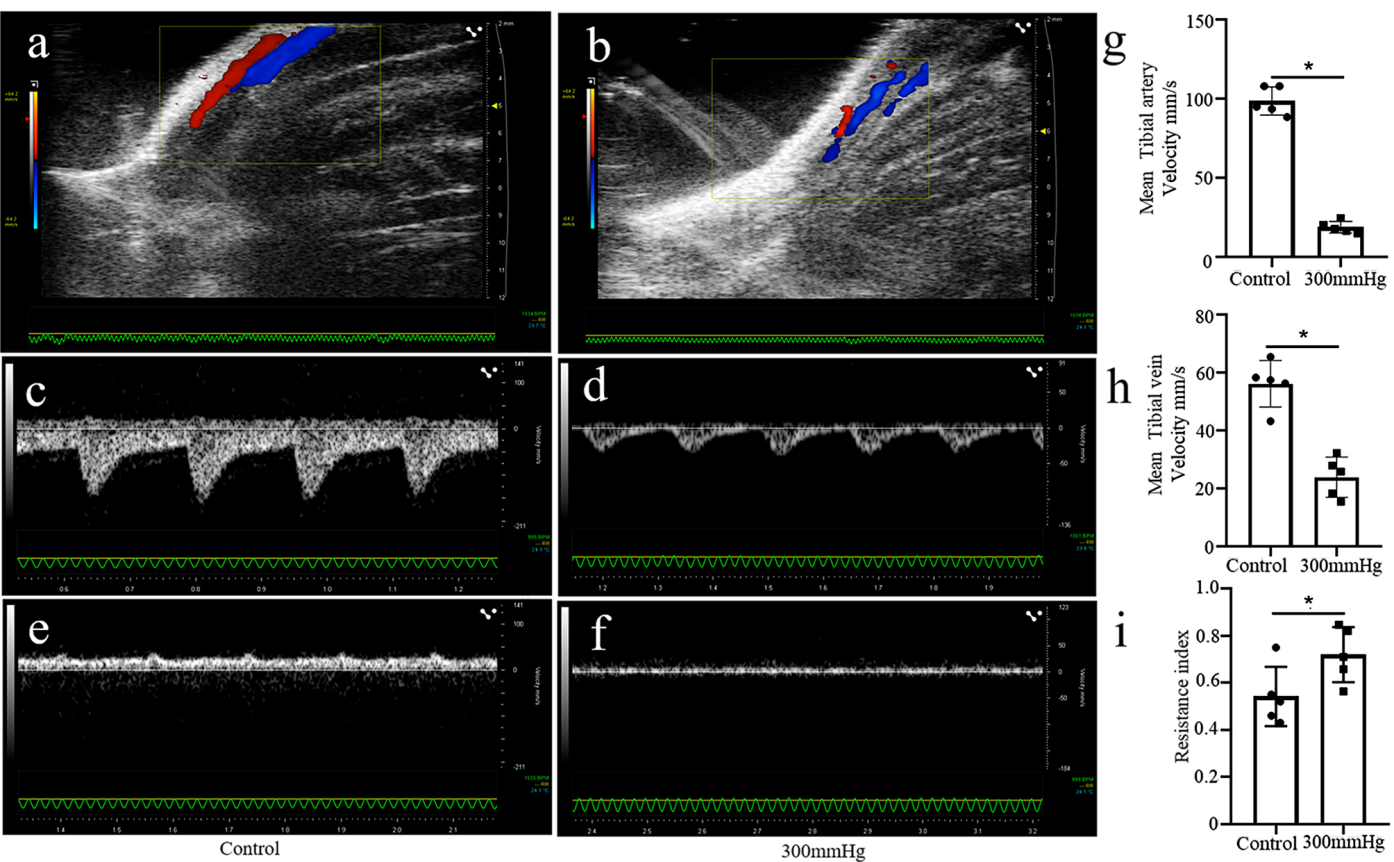
Lower limb blood flow following combined injury on the lower leg of rats. (**a, b**) Representative images of PW Doppler for the tibial arteries and veins of the rats. (**c-f**) Representative images of PW Doppler for the tibial artery and vein velocity. (**g, h**) Statistical analysis of mean tibial artery and vein velocity. (*n* = 5 for each individual group) (i) Statistical analysis of RI. (*n* = 5 for each individual group). The data are presented as the mean ± SD. *P < 0.05. PW Doppler, pulsed-wave Doppler; SD, standard deviation

It was well known that the blood flow would gradually decrease with the increase of ICP in ACS. Previous studies reported that Color Doppler Ultrasound was widely used in hemodynamic detection [30, 31]. Hence, we examined the blood flow of lower leg in 300 mmHg combined injury group using color Doppler ultrasound (Fig. 5a, b). As shown in Fig. 5c-h, mean tibial artery and vein velocities were significantly decreased in the 300 mmHg combined injury group compared with the control group. Furthermore, RI was significantly increased in the 300 mmHg combined injury group. These data further indicated that combined injury reduced the blood flow in the lower limb.

## Discussion

Previous study defined ACS as the dysfunction of tissue circulation within the fascia compartment due to increased pressure [1]. However, the diagnosis and treatment of ACS is still under debate or controversial for decades. It is of great importance to study the pathological molecular mechanisms of ACS based on animal model for the diagnosis and prognosis of ACS. Previous studies had attempted to develop ACS animal models, however, these models are not ideal. In this study, we creatively combined blunt trauma injury with compression injury and successfully developed a novel ACS model with delta P measurement of less than 30 mm Hg. The current combined injuries ACS model reflected the classic diagnosis criteria, including changes in ICP, ΔP, serum biochemistry, pathology and lower limb blood flow. All these data indicated that moderate blunt trauma combined with compression of the lower leg can mimic the ACS-like injury.

Abnormally increased fascia compartment pressure caused by various factors may lead to the ACS. Microvascular injury at the initial impact site may results in extravasation of blood and increased interstitial fluid pressure [2]. The restraint of the fascia can cause an increase in the perfusion pressure of the tissue in the fascia compartment, resulting in tissue hypoxia. Tissue hypoxia may leads to cellular edema, which further aggravates tissue hypoxia, thus forming a vicious cycle [24]. The two animal models of intra-fascial perfusion and intra-fascial balloon can flexibly control intra-fascial pressure and can play an important role in studying the pressure threshold of ACS induced muscle necrosis [4, 6, 7, 12]. However, neither of the two models is suitable for studying the pathogenesis of ACS. Reduced fascial compartment perfusion may cause ACS, but it does not mean there is no perfusion. Previous studies have used tourniquets or ligation of blood vessels to block blood perfusion of tissue to simulate ACS-induced injury [15, 18, 19, 32, 33]. However, this confuses ischemia-reperfusion injury with trauma-induced ACS-like injury. Our study could well simulate clinical trauma-induced ACS-like injury to create an ACS animal model with a Δ*P* value of less than 30 mmHg.

Trauma is an important causal factor of ACS. Deep fascia wraps around muscle bundles and connects them to bone and tendon. Rigid fascia binding to swollen tissue in the fascia compartment can lead to a vicious cycle of cellular edema and hypoxia. In this study, different degrees of blunt trauma (0.5 kg, 1.0 kg, 2.0 kg) to the lower legs of rats alone were attempted to simulate ACS model. However, none of the three trauma injury groups had Δ*P* values of less than 30mmHg (Fig. 1d; Table 1). This suggested that blunt trauma alone could not mimic ACS-like injury. Interestingly, the 2.0 kg blunt injury rats had the severe damage, with a lower ICP. This may be related to the destruction of the fascial compartment structure after the open injury, and the pressure in the fascial compartment did not reach the level necessary for ACS diagnosis, which may be related to the compliant dissipation of stress through the rat fascia. Further experiment with moderate energy blunt trauma, compression of the lower legs of rats with a modified compression device mimicked the tight binding of facia around the muscle in ACS. The swelling of the lower legs of the rats became more severe as the pressure increased, and tension blisters appeared in ankle and plantar of some rats after ACS formation in the 300 mmHg and 400 mmHg compression pressure groups. In clinical practice, tension blisters often occur on ankles, knees, elbows, etc.as a result of severe soft tissue injury and subsequent edema [34]. This phenomenon also reflects the high pressure of the fascia compartment in this ACS-like injury model. Although there is still no study confirming the causal relationship between ACS and tension blisters, many studies have reported that ACS can be combined with blisters [35–37]. Hou et al. indicated that tension blister is a phenomenon through which the pressure from intra-compartmental was released, and that blister formation could be a sign of pressure release, leading to remission of ACS symptoms [38]. To the best of our knowledge, this is the first report to simulate the symptoms of tension blisters formation in ACS animal models, providing theoretical foundation for future researches on the pathophysiology of ACSrelated tension blisters. However, only about 70% of ACS rats developed blisters in the 300mmHg and 400mmHg compression groups, and we speculate that this phenomenon may be related to individual differences. In future studies, inbred rats will be used to further investigate the relationship between tension blisters formation and ACS.

Orrapin et al. found that serum CK, CKMB, LAC, LDH, ALT and AST were correlated with ACS [39]. Another study found significant increases in serum creatinine and urea nitrogen levels in ACS patients [40]. This study confirmed that these serum analytes were significantly increased in this rat ACS model. Until now, there have not been specific and sensitive biomarkers for the diagnosis of ACS. Both intra-compartmental infusion and balloon tourniquet models of ACS relied on artificial ischemia. However, this study demonstrated that spontaneous limb ischemia was caused by limb swelling. Doppler ultrasound further confirmed that the mean arterial and venous blood flow velocities decreased and the arterial RI increased due to the hind limb swelling, which was consistent with the results of another pulse-Doppler study simulated compartment syndrome [30].

This study has several limitations. Firstly, the number of animals used in this study was small so it was difficult to find out the exact relationship between ACS and blister formation. Secondly, there are differences in the anatomy of limbs between rats and human. Larger animals such as pigs and sheep may be more anatomically similar to humans. Kalns et al. [11, 12]. showed that the calf fascia of pig, similar to human, was inelastic and could not withstand massive swelling, whereas the fascia of rodent could withstand massive swelling without increasing pressure in the fascial compartment. Further research is needed to explore whether anatomic differences associated with small animals may adversely affect the studies on compartment syndrome. Therefore, the benefits of anatomical similarity and the research purpose of the experiment must be considered when selecting experimental species. Finally, as Heckman et al. suggested, the various ACS animal models led to a lack of consensus on the recommended pressure thresholds for ACS diagnosis. In this study, the pressure threshold of ΔP < 30 mmHg was selected as the criterion for successful ACS modeling, similar to the criterion for human ACS diagnosis. In addition, invasive manometry was used to continuously monitor the intra-compartmental pressure of the rats’ lower legs, which would add the additional trauma to the rats. This may affect the accuracy of the ICP. Future studies could focus on developing an alternative ICP monitoring device, such as a miniature implantable manometry.

## Conclusion

We have established a novel rat model of ACS with Δ*P* value of less than 30 mmHg, which could better simulate the pathophysiological processes of the occurrence and development of ACS in human body. In the future, this model could be used to investigate the more complex cellular and genetic mechanisms underlying of ACS, further driving the development of new diagnostic and therapeutic approaches.

## Data Availability

The data used or analyzed in this study are available from the corresponding author upon reasonable request.
